# O.6.2-9 Beyond human and public health: a comprehensive analysis of different health concepts in physical activity promotion

**DOI:** 10.1093/eurpub/ckad133.279

**Published:** 2023-09-11

**Authors:** Roberto Galindo, Jantje Ammermann, Fabio El Kaddouri García, Peter Gelius, Karim Abu-Omar

**Affiliations:** Friedrich-Alexander-Universität Erlangen-Nürnberg, Germany; roberto.galindo@fau.de; Friedrich-Alexander-Universität Erlangen-Nürnberg, Germany; roberto.galindo@fau.de; Friedrich-Alexander-Universität Erlangen-Nürnberg, Germany; roberto.galindo@fau.de; Friedrich-Alexander-Universität Erlangen-Nürnberg, Germany; roberto.galindo@fau.de; Friedrich-Alexander-Universität Erlangen-Nürnberg, Germany; roberto.galindo@fau.de

## Abstract

**Background:**

There is a growing focus on health topics that go beyond individual and public health and attempt to grasp the relationship between the environment, animal and human health.

Currently little is known about how these holistic perspectives are being integrated in the field of Physical activity (PA) and PA promotion. The aim of our systematic review[AJ1] is to present the current state of research regarding PA and the holistic perspectives, including their evolution throughout time, their frequency of appearance in the literature and how they are being utilized in PA related research.

**Methods:**

We conducted a comprehensive search using PubMed, Scopus, and Web of Science searching for PA, its synonyms and permutations in combination with six previously identified holistic health concepts:

(1) Planetary Health, (2) environmental health, (3) One Health, (4) Eco Health, (5) Geo Health, and (6) Gaia. The search resulted in a total of 924 articles after duplicates were removed. Three independent researchers screened title/abstract and resulting full-texts using Covidence. Results were analysed regarding the frequency of each concept and evaluating the extend of how the health concepts are utilized and implemented in PA promotion.

**Results:**

The screening process resulted in 52 articles that met the inclusion criteria and focused on PA and its promotion in combination with one of the six health concepts. Preliminary results show an increased number of publications regarding holistic health concepts that go beyond human health in the field of PA promotion and its research in recent years.

Results indicate that only few studies implement these health concepts but rather only integrate the terms without its application. Further, the statistical evaluation shows that the concept of environmental health is the most frequently adopted health perspective followed by planetary health.

**Conclusion:**

The identified articles show that the holistic health perspectives gain more relevance in the field of PA promotion. However, there are still only a few studies that apply these health concepts in the field of PA promotion. Further research needs to be conducted on the integration of different health concepts that go beyond human health in the field of PA promotion.

